# Neutrophil extracellular traps in pulmonary fibrosis: mechanisms, immunity and therapeutic targets

**DOI:** 10.3389/fimmu.2026.1765590

**Published:** 2026-03-04

**Authors:** Jing Wu, Yongbin Hu

**Affiliations:** 1 Department of Pathology, Basic Medical School, Central South University, Changsha, China; 2 Department of Pathology, Xiangya Hospital, Xiangya School of Basic Medical Sciences, Central South University, Changsha, China

**Keywords:** immunity, inflammation, IPF, NETosis, NETs, neutrophil extracellular traps, pulmonary fibrosis

## Abstract

Pulmonary fibrosis, a heterogeneous and fatal interstitial lung disease, lacks curative therapies and specific biomarkers, posing great clinical challenges. Neutrophil extracellular traps (NETs) are key inflammatory mediators in pulmonary fibrosis pathogenesis, yet subtype-specific regulatory mechanisms and targeted therapeutic optimization remain unclear. This review systematically elucidates the distinct NETosis pathways across various subtypes. We further elaborate the multi-layered mechanisms of NETs in mediating inflammation-fibrosis transition, fibroblast activation, and innate-adaptive immune crosstalk, revealing subtype-specific pathological effects of NETs in pulmonary fibrosis. Additionally, we conduct a critical comparison of three NET-targeted therapeutic strategies and their advantages, limitations as well as subtype adaptability. Finally, we summarize the clinical transformation challenges of NET-targeted therapies and propose optimization directions. This review provides a precise theoretical framework for understanding PF immunopathogenesis and offers actionable insights for advancing NET-targeted precision medicine in pulmonary fibrosis.

## 1 Pulmonary fibrosis and innate immune dysregulation

### 1.1 Clinical and pathological background of pulmonary fibrosis

**Figure 1:**
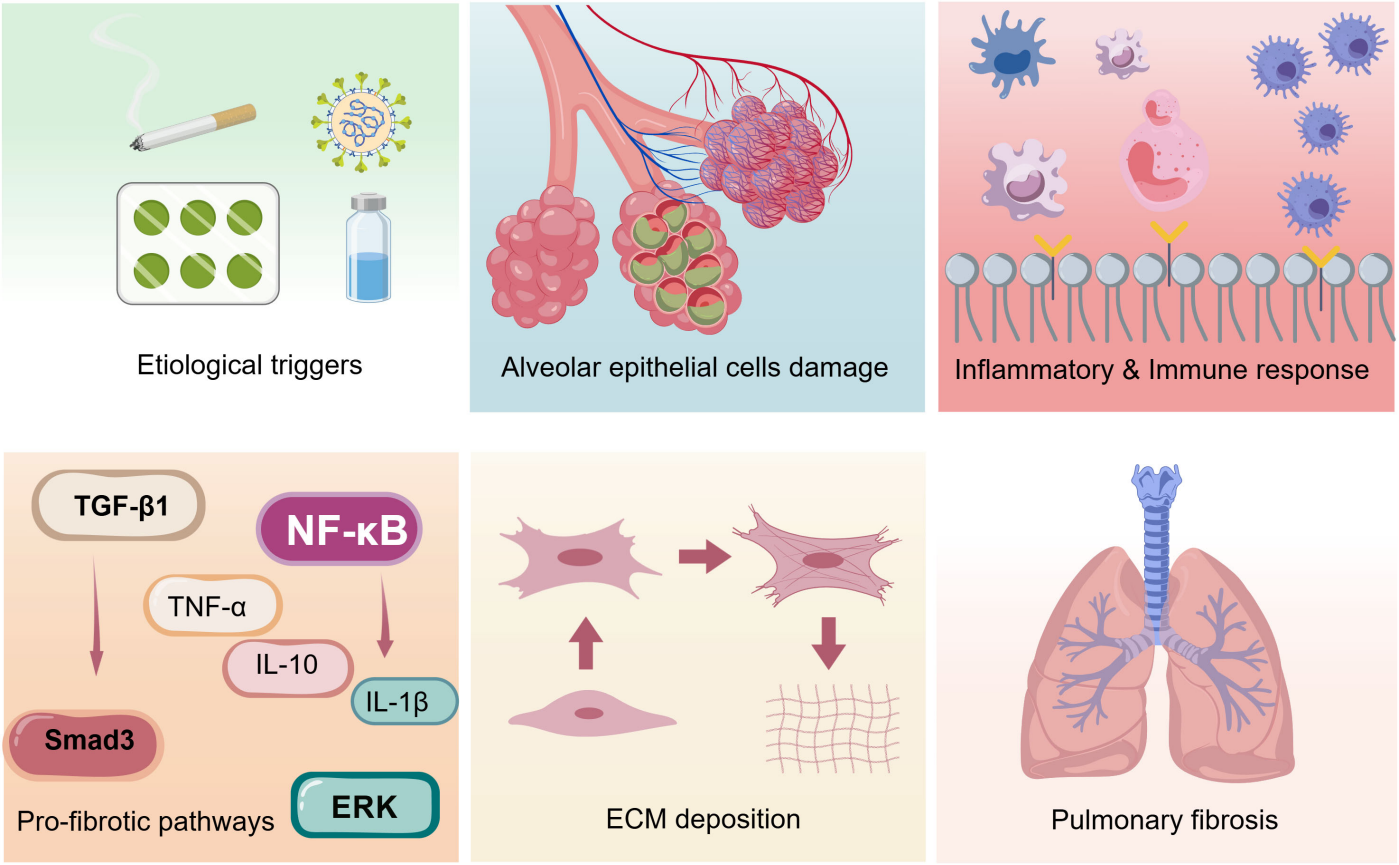
Fibrosis processes induced by different etiologies. Schematic representation of the core fibrotic programs triggered by distinct pathogenic stimuli, highlighting key cellular responses and molecular events that drive extracellular matrix accumulation and tissue fibrosis.

Pulmonary fibrosis (PF) refers to a heterogeneous group of chronic, progressive, and irreversible interstitial lung diseases (ILDs) (1, 2). The pathological characteristics of PF are diffuse interstitial inflammation, structural destruction of lung tissue, and pulmonary parenchymal fibrosis, which ultimately leads to irreversible loss of lung function, and the clinical manifests including reduced lung compliance, impaired gas exchange, progressive dyspnea, and even respiratory failure (1, 3, 4). When the lungs are damaged, under the continuous action of damaging stimuli, lung tissue is repeatedly damaged, excessive deposition of extracellular matrix (ECM) components (including collagen, fibronectin, and proteoglycans) in the alveolar spaces and pulmonary interstitium, accompanied by abnormal activation, proliferation and differentiation of fibroblasts (4–6). PF encompasses a spectrum of disorders with distinct etiologies, including idiopathic pulmonary fibrosis (IPF), cystic pulmonary fibrosis (CF), asthma, silicosis, chronic obstructive pulmonary disease (COPD), etc. (1, 3, 7). Among them, IPF is the most common and severe form with unknown pathogenesis, and secondary PF associated with multiple factors, including, aging, environmental factors, toxic, autoimmune, drug-induced, infectious, or traumatic injuries (1, 3, 7) (**Figure 1**).

Currently, the prevalence of PF is on the rise worldwide, imposing a heavy disease burden with a notably high case fatality rate (8, 9). Despite the application of current antifibrotic therapies, the disease still carries a poor prognosis, with a median survival time of merely 5 to 7 years following diagnosis (10).

The clinical challenges associated with pulmonary fibrosis (PF) are mainly manifested in four key aspects: Firstly, the early clinical manifestations of PF are non-specific, which predisposes to misdiagnosis and thus delays the implementation of targeted therapeutic interventions (11, 12). Secondly, there is still no curative treatment for PF to date, and the available clinical medications only exert a disease-modifying effect to slow down its progressive course (13). Thirdly, the lack of sensitive and specific biomarkers has become a major bottleneck restricting the early diagnosis of PF (14). Fourthly, the clinical management of PF is further complicated by the presence of various comorbidities in affected patients (15).

### 1.2 Pathophysiological differences among pulmonary fibrosis subtypes

Clinically, the currently recognized global classification system categorizes pulmonary fibrosis (PF) into two major subtypes: idiopathic pulmonary fibrosis (IPF) and non-idiopathic pulmonary fibrosis (non-IPF-PF) (1, 10). IPF represents the most common, typical and well-characterized subtype in clinical practice and basic research (16). By contrast, non-IPF-PF refers to fibrotic lesions with identifiable etiologies or triggering factors, encompassing cases induced by occupational exposure, underlying systemic diseases, pharmaceutical agents and physical-chemical insults (7).

Notably, these subtypes exhibit distinct core pathological features. IPF is pathologically defined by usual interstitial pneumonia (UIP) pattern, featured by patchy interstitial fibrosis, alternating fibroblastic foci, honeycomb lung formation, and minimal inflammatory cell infiltration (16). For environmental/occupational exposure-related PF—exemplified by silicosis fibrosis—the disease is initiated by long-term inhalation of free silica dust, with pathological hallmarks including pulmonary interstitial dust deposition, silicotic nodule formation, and nodule-centric progressive interstitial fibrosis, accompanied by prominent neutrophil-predominant inflammatory infiltration and extensive collagen deposition (17). Disease-associated PF displays heterogeneous pathological characteristics that vary with the primary disorder, and lacks the typical UIP pattern (1). PF induced by drugs or physical-chemical factors is predominantly characterized by diffuse alveolar and interstitial lung injury (18). In addition, recent research in the PF field have reported that progressive fibrotic interstitial lung disease (PF-ILD) characterized by persistent, progressive pulmonary fibrotic progression and a concomitant progressive decline in lung function (19).

The core pathological distinctions between IPF and non-IPF-PF subtypes are manifested in two key aspects: First, the immune microenvironment profiles differ substantially—IPF is characterized by a mild inflammatory infiltrative immune microenvironment, whereas non-IPF-PF features a robust inflammatory state with prominent infiltration of neutrophils and other inflammatory cells. Second, the rates of fibrotic progression vary: IPF typically presents with slow, progressive interstitial remodeling, while fibrotic progression in non-IPF-PF is closely coupled to the activity of the underlying primary disease (1, 16, 17).

### 1.3 Neutrophil extracellular traps and innate immune mechanism

In the human body, neutrophils are involved in innate immunity and adaptive immunity, and can interconnect and interact with a variety of immune cells, which is the first line of the body’s defense (20). NET is one kind of complex released by neutrophil that made up of DNA and a variety of granule proteins and peptides (21). These proteins and peptides include citrullinated histone (Cit-H3), neutrophil elastase (NE) and myeloperoxidase (MPO) etc. and them can be important markers and detection indicators in the detection of NET (22, 23).

**Figure 2:**
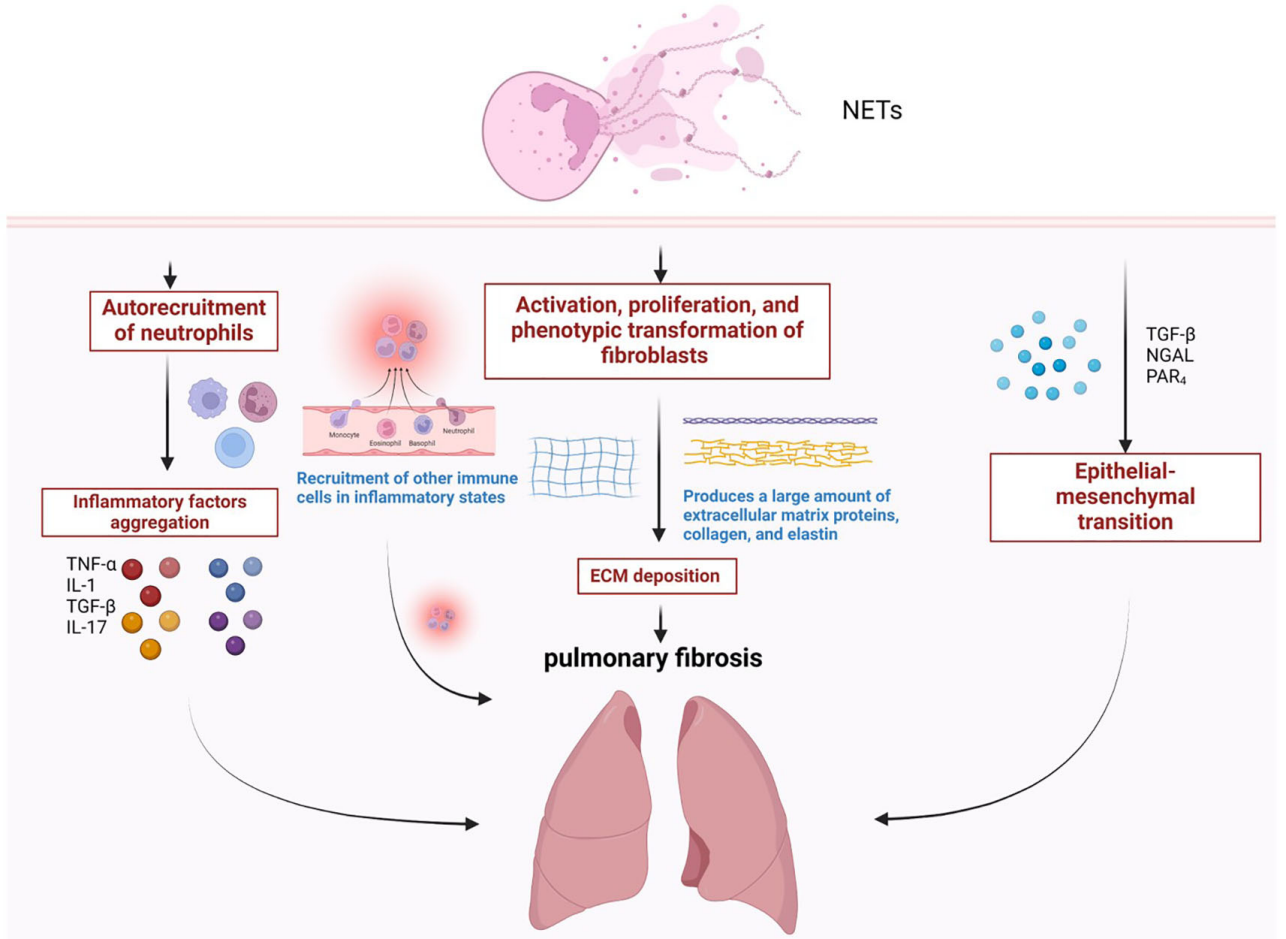
The role of NETosis in the process of pulmonary fibrosis. **(a)** The imbalance in the release of NETs leads to the recruitment of neutrophils and the secretion of a variety of inflammatory factors. **(b)** NETs can promote the proliferation and migration of fibroblasts, and promote the differentiation of fibroblasts into myofibroblasts, causing excessive deposition of ECM. **(c)** Neutrophils can effectively induce epithelial-mesenchymal transition (EMT) through NETosis.

At the site of inflammation, neutrophils release large numbers of NETs, which surround the invading pathogen and help leucocytes clear and engulf pathogens and other microorganisms (21). However, the overexpression of NETs will cause tissue damage and inflammation progress (24). Recent studies have shown that abnormally activated NETs can be used as key inflammatory mediators to participate in the pathological process of chronic inflammatory and fibrotic diseases by releasing pro-inflammatory factors, inducing cell activation, and damaging the tissue microenvironment (25). By blocking the formation of NETs or promoting the clearance of NETs, the progression of inflammation and tissue fibrosis can be effectively suppressed (23, 26). Therefore, we consider that NETs may be significant and vital target for investigate the mechanism of pathology, developing drugs and treating pulmonary fibrosis (**Figure 2**).

However, the crosstalk between NETs and fibroblasts/immune cells, potential NET-targeted therapeutic strategies, and the associated risks remain largely elusive—key questions that this review seeks to systematically clarify and address.

## 2 NETosis and its dominant pathway in the PF microenvironment

### 2.1 Three core pathways of NETosis

The formation of NETs is generally called NETosis and it’s actually a novel mode of death that distinguished from apoptosis and necrosis. There are three way of formation of NET according to different stimuli (21).

The first way, known as suicidal NETosis, is dependent on the involvement of NADPH oxidase and Raf/MEPK/ERK signaling pathway. This pathway causes plasma membrane rupture and cell death (23, 27). Neutrophil under the stimulation of phorbol ester (PMA), interleukin-1β (IL-1β) or others to activate protein kinase C (PKC) (28). NADPH oxidase will activated by Raf/MEPK/ERK signaling pathway and then stimulate the generation of reactive oxygen species (ROS) and Cit-H3 (27). The final cause rupture of nuclear membrane and release of NETs. On the contrary, the second way is vital NETosis, which independent on the involvement of NADPH and Raf/MEPK/ERK signaling pathway (29). The pathological feature of the formation is relatively intact nuclear membrane and cell membrane, and the activity and function of neutrophil are maintained (30). Some new studies of the third type of NETosis in recent years and it occur when neutrophil stimulated by Lipopolysaccharides (LPS) or Granulocyte-macrophage colony-stimulating factor (GM-CSF) (30, 31).

### 2.2 The pulmonary fibrosis microenvironment-mediated regulation of the NETosis pathway

#### 2.2.1 IPF: mitochondrial ROS and vital NETosis as dominant pathways

IPF is characterized by low inflammation, high fibrosis, and slow progression, without clear exogenous stimuli, and lung tissue has been in a microenvironment with a large amount of ECM deposition and mild infiltration of inflammatory cells for a long time, which cannot meet the strong ROS burst conditions required by classical NOX-dependent NETosis (29, 32, 33).

Vital NETosis is key pathway for the sustained release of NETs in IPF, and a large number of dense ECMs in IPF lung tissue anchor neutrophils, mediating PAD4 activation by activating calcium influx, allowing neutrophils to continue to release NETs while maintaining a viable state, providing a continuous signal for fibroblast activation (32–34). The study found that the number of ECM-neutrophil complexes in IPF lung tissue was higher than that of healthy people, and blocking ECM integrin reduced NETs release while significantly reducing the expression level of fibroblast α-SMA, confirming that this pathway is directly related to the progression of fibrosis in IPF (34–36).

In addition, quantitative studies have shown that mitochondrial DNA (mtDNA) levels in bronchoalveolar lavage fluid (BALF) in IPF patients are higher than those in healthy individuals, while autophagy inhibitors can significantly reduce the release of NETs from IPF-derived neutrophils, and mitochondria-targeting antioxidant MitoQ can reduce NETs-associated collagen deposition, further confirming the central role of this pathway in IPF (37–40).

It is worth noting that classical NOX-dependent NETosis have a weak effect in IPF and the expression of NOX2 mRNA in lung tissue of patients with stable IPF is only lower than silicosis patients, suggesting that this pathway is not the dominant pathway for IPF (41–43).

#### 2.2.2 Non-IPF-PF: NOX-dependent NETosis and NLRP3 inflammasome as dominant pathways

Non-IPF-PF, represented by silicosis, are characterized by strong inflammation, clear etiology and rapid progression (17). And the NETosis dominant pathway focuses on inflammation amplification and rapid fibrotic transformation, dominated by classical NOX-dependent NETosis and NLRP3 inflammasome-NETs positive feedback loop, which is in stark contrast to IPF (41–44).

Classical NOX-dependent NETosis is the core priming mechanism of early inflammatory outbreaks in silicosis (43). And silica dust can directly activate NOX1/NOX2 on the surface of neutrophils, triggering ROS bursts, and then synergistically activating PAD4, NE, and MPO, driving chromatin depolymerization and NETosis (42). Studies showed that the level of Cit-H3 in silicosis mice was increased compared with the control group, and NOX1 inhibitors reduced silica dust-induced release of NETs and lung tissue damage (45, 46). The degree of silicosis fibrosis in PAD4 knockout mice was reduced compared to wild-type mice, which fully confirms the central role of this pathway (47). In addition, exogenous stimulants can simultaneously drive NETosis and lung epithelial cell PANoptosis through the NOX1/ROS axis, releasing damage-associated molecular patterns (DAMPs) and forming an inflammatory vicious cycle (48–50).

NLRP3 inflammasome-NETs positive feedback loop is a key amplifier of silicon pneumonitis-fibrosis transformation, silica dust can activate NLRP3 inflammasomes in macrophages, promote IL-1β release, and IL-1β can further promote neutrophil activation and NETosis, forming a sustained inflammatory amplification effect (44). Studies showed that the levels of NETs in silicosis lung tissue of NLRP3 knockout mice were reduced compared with wild-type mice (44, 51). IL-1β neutralizing antibodies effectively block this positive feedback loop and reduce collagen deposition (52).

## 3 Pathological mechanism of NETs in pulmonary fibrosis

### 3.1 NETs mediate the inflammation-fibrosis transition

The core pathological feature of pulmonary fibrosis is the aberrant conversion of early inflammatory responses to late-stage irreversible fibrosis, with NETs acting as key regulators driving this process (53). NETs bridge the inflammation-fibrosis transition via three core nodes. First, NE, MPO, and other components released by NETs directly damage pulmonary epithelial cells, induce the infiltration of innate immune cells, and trigger the early inflammatory cascade (54, 55). Second, NETs modulate the polarization of immune cells toward profibrotic phenotypes (e.g., M2 macrophage polarization), shift the inflammatory microenvironment from an anti-inflammatory to a profibrotic state, and drive the secretion of fibrosis-related factors such as TGF-β1 and IL-17 (55–57). Third, NETs crosslink with ECM to form stable NET-ECM complexes; these complexes not only impede inflammatory resolution but also provide sustained microenvironmental signals for fibroblast activation, ultimately facilitating the irreversible conversion of inflammatory responses to fibrosis (56, 58, 59). In summary, NETs are not merely inflammatory mediators but core bridging molecules linking early inflammation to late-stage tissue remodeling in pulmonary fibrosis.

### 3.2 Crosstalk between NETs and fibroblasts

**Figure 3:**
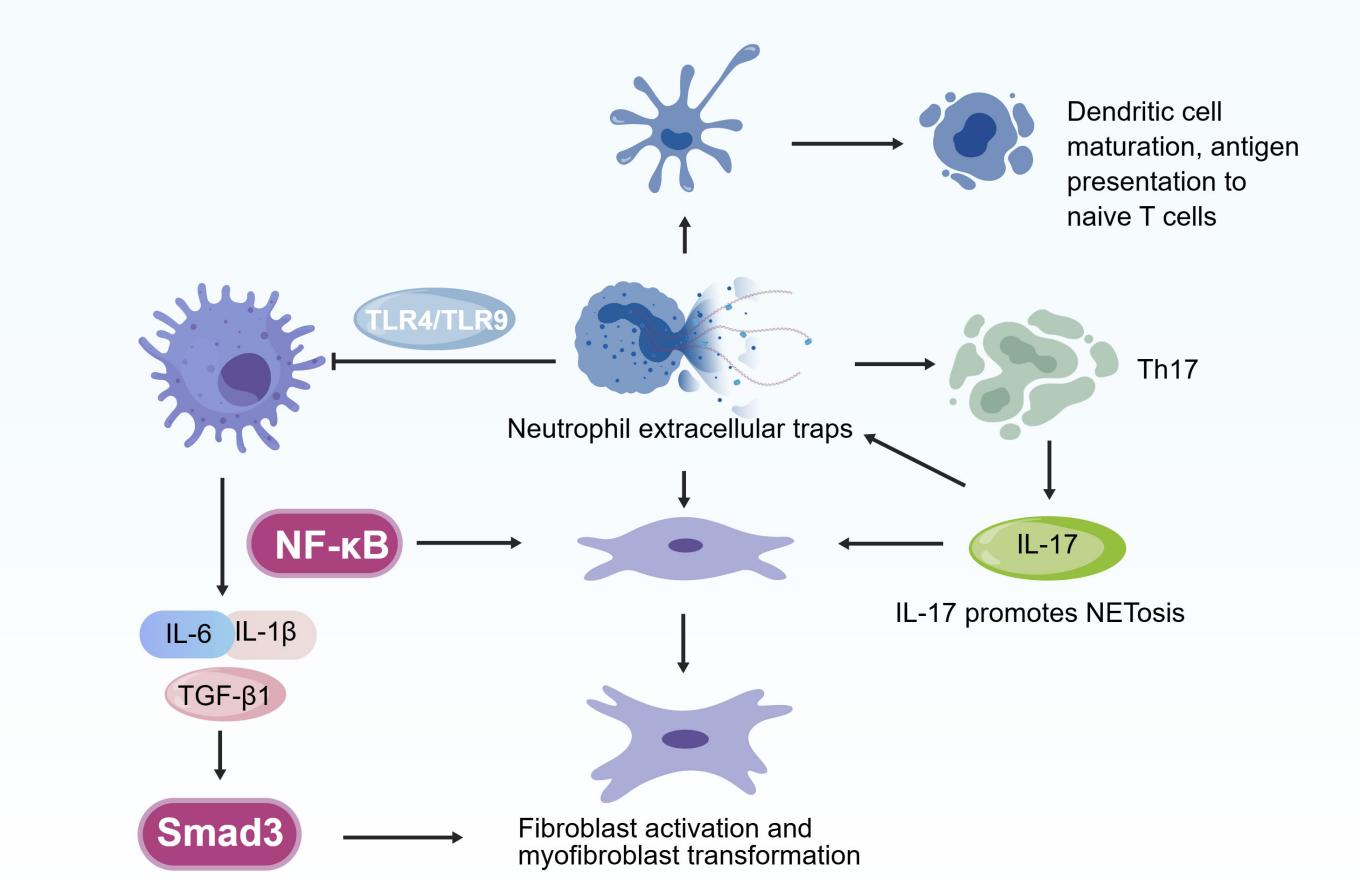
Crosstalk between NETs and fibroblasts. NETs not only can bind directly to fibroblast surface receptors to initiate activation signals, but also indirectly activate fibroblast signaling pathways by regulating immune cell (e.g., Dendritic cell, Th17, macrophage) secretion of profibrotic cytokines.

The central target of NETs to achieve bridging is fibroblasts. It precisely drives fibroblast activation and ECM deposition through direct contact and indirect signal regulation, and the specific mechanism is as follows (53, 58) (**Figure 3**).

On the one hand, NETs can bind directly to fibroblast surface receptors to initiate activation signals. In many related studies, it has been reported that NETs bind TLR9 receptors and RAGE receptors on the surface of fibroblasts, activating NF-kB pathway within fibroblasts, thereby promoting the secretion of pro-inflammatory factors (such as IL-6) and the deposition of ECM (53, 60, 61).

On the other hand, NETs indirectly activate fibroblast signaling pathways by regulating immune cell secretion of profibrotic cytokines. First, NETs induce macrophages to polarize toward the M2 phenotype, and M2 macrophages secrete TGF-β1. This cytokine binds to TGF-β receptors (TGF-βR) on the fibroblast surface, initiates Smad3 phosphorylation, and promotes fibroblast activation as well as collagen I/III synthesis (62–64). Second, NETs-mediated immune networks induce Th17 cells to secrete IL-17, and this cytokine binds to IL-17 receptors (IL-17R) on fibroblasts, activates NF-κB nuclear translocation, and enhances fibroblast proliferation and α-SMA expression (65, 66).

### 3.3 Specific roles of NETs in pulmonary fibrosis subtypes

The mechanisms by which NETs drive pulmonary fibrosis exhibit marked heterogeneity across distinct pulmonary fibrosis subtypes, owing to differences in their immune microenvironment, etiology, and disease progression characteristics (67). NETs display subtype-specificity in their functional targets, core mechanisms, and effector intensity, as follows.

First, idiopathic pulmonary fibrosis (IPF) is characterized by low-grade inflammation, prominent fibrosis, and slow progression (1). The pathological effects of NETs in IPF are dominated by direct actions on fibroblasts and indirect effects mediated via the TGF-β1/Smad3 pathway (56). Given the mild inflammatory cell infiltration in IPF lung tissue, NETs do not depend on a robust inflammatory microenvironment, instead, they bind directly to fibroblasts via TLR9/RAGE receptors and synergize with M2 macrophage-secreted TGF-β1 to augment fibrotic effects (60, 61, 64). Additionally, NETs form complexes with dense ECM in IPF lung tissue, which acts as a persistent driver of fibrosis and constitutes a key underlying cause of the irreversible progression of IPF (58).

Second, non-IPF-PF, represented by silicosis, are characterized by robust inflammation, rapid progression, and a well-defined etiology (1). The pathological effects of NETs in this subtype are primarily mediated by the inflammation-fibrosis transition and indirect activation of the IL-17/NF-κB pathway (66, 68). Exogenous stimuli such as silica dust directly activate neutrophils to release NETs, provoking a vigorous early inflammatory response. NETs then rapidly drive the conversion of inflammation to fibrosis by augmenting the pro-fibrotic bias of the inflammatory response (54). Concurrently, NETs drive robust Th17 cell proliferation, which sustains the amplification of both inflammatory and fibrotic effects (69). Furthermore, NETs can bind to silica dust particles to form NET-dust complexes, thereby prolonging inflammatory stimulation and accelerating fibrotic progression (70).

## 4 NETs modulate the immune network in pulmonary fibrosis

### 4.1 NETs modulate the innate immune cells

As key innate immune inflammatory mediators, NETs directly or indirectly regulate the functions of macrophages and DCs, and the crosstalk between neutrophils and these two cell types further amplifies NETs production. NETs activate macrophages to release inflammatory factors, which in turn promotes neutrophil activation and NETosis (71). Meanwhile, NETs promote DC maturation and migration to lymph nodes and thus triggering subsequent adaptive immune activation (72, 73). DNA-Cit-H3 complexes bind to TLR4/TLR9 on the macrophage surface, activating the NF-κB pathway and inducing macrophages to secrete abundant pro-inflammatory factors (IL-6, IL-1β) and pro-fibrotic cytokines (TGF-β, IL-10), thereby initiating a pro-fibrotic inflammatory microenvironment and driving fibroblast activation (56, 74–76).

### 4.2 NETs mediate adaptive immunity

Mature DCs present NET-associated antigens to naïve T cells, and NETs directly activate T cell receptors via DNA-LL37 complexes, lowering T cell activation thresholds and selectively inducing Th17 cell differentiation. IL-17 secreted by Th17 cells in turn fuels this vicious circle (65). IL-17 not only promotes neutrophil activation and NETosis, but also directly activates the IL-17R-NF-κB pathway in fibroblasts to enhance their proliferation and ECM synthesis (66, 77, 78). This crosstalk is notably pronounced in autoimmune disease-associated PF and silicosis, serving as a key driver of sustained inflammatory amplification and accelerated fibrotic progression (79).

### 4.3 Synergistic effects in immune networks

Innate-adaptive immune crosstalk ultimately forms a fibrosis-promoting closed-loop network via two pathways. First, various immune cell-secreted cytokines synergistically amplify profibrotic signals, encompassing multiple steps in fibroblast activation (77). Second, NETs co-bind with immune cell-secreted cytokines to fibroblast surface receptors, activating downstream signaling cascades (60). The core feature of this network is the sustained amplification of fibrotic signals, and elevated NETs sustain long-term profibrotic effects through this immune network (80).

## 5 Therapeutic strategies for pulmonary fibrosis targeting NETs

### 5.1 Classification and critical comparison of therapeutic strategies

The detrimental effects of NETs on the fibrotic pathological process stem from an imbalance between NET formation and clearance (80). Excessive NET release coupled with impaired clearance leads to their aberrant accumulation at the site of tissue injury. Such abnormal accumulation triggers the massive release of proinflammatory cytokines, which on the one hand act on various immune cells (e.g., macrophages), and on the other hand activate multiple intracellular signaling pathways; this consequently drives the progression of pulmonary fibrosis and causes repeated damage to pulmonary tissue cells. Therefore, current research into NET-targeted therapeutic strategies for pulmonary fibrosis focuses on three key approaches: 1) Inhibition and blockade of NETosis. 2) Elimination of preformed NETs. 3) Blockade of NET-associated signaling pathways (**Table 1**).

**Table 1:** Classification and critical comparison of therapeutic strategies.

Therapeutic strategy	Representative agents	Advantages and subtype adaptability	Risks and limitations
Inhibition of NETosis	1.PAD4 inhibitors 2.NE inhibitors 3.NOX inhibitors 4.CDK4/6 inhibitors	1.Directly block NETs generation 2.Optimal for early stage PF	1.Increased infection risk 2.No clearance of preformed NETs 3.Limited efficacy in advanced PF
Elimination of Preformed NETs	1.DNase I 2.Annexin A1 3.Anti-Cit-H3 antibodies	1.Low infection risk 2.Suitable for advanced PF 3.High targeting specificity	1.DNase I has poor dense ECM penetration 2.No prevention of *de novo* NETs formation 3.anti-Cit-H3 antibodies with high cost and poor adherence
Blockade of NET-Associated Signaling Pathways	1.TGF-β1/Smad3 inhibitors 2.NF-κB inhibitors 3.TLR9/RAGE inhibitors 4.IL-17/IL-17R antibodies	1.Directly cut off profibrotic signals 2.TLR9/RAGE/TGF-β1/Smad3 for IPF 3.NF-κB/IL-17 for non-IPF-PF	1.Impairs normal cellular signaling 2.Pan-targeted inhibitors with off-target effects 3.Single-pathway blockade triggers pathway compensation

#### 5.1.1 Inhibition and blockade of NETosis

The pathological effects of NETs are contingent upon their generation and release into the lung tissue microenvironment. Thus, blocking NETosis represents the most direct upstream source-blocking strategy. This approach inhibits NETs release by targeting key molecular mediators of NETosis (e.g., PAD4, NE), thereby abrogating their sustained activation of the inflammatory-fibrotic axis from the upstream. Representative agents include selective PAD4 inhibitors and NE inhibitors.

First, since the main components of the NET structure include neutrophil proteases, the use of neutrophil protease inhibitors can impair neutrophil migration and phagocytosis, which can further prevent neutrophil recruitment and activation, and reduce the release of NETs (23, 81). And neutrophil elastase plays a role in decoagulation of chromatin during NETosis (23). As a result, the release of NETs can be reduced, thereby reducing cytotoxic effects and pathogen escape (82). Previous studies have found that the respiratory tract of CF patients secretes a large amount of neutrophil protease, which is an important secretagogue of the respiratory tract, and NE inhibitors can block this secretion response (83, 84). Second, PAD4 catalyzes histone citrullination, promoting chromatin decoagulation during NET formation (22). PAD4 inhibitor can block the formation of citrullinated histones, thereby influencing the NETosis and promoting the balance of NETs formation and clearance (85). Previous studies have shown that PAD4 inhibitor can reduce NET levels and significantly reduce NETosis in a variety of inflammatory diseases (86, 87). Therefore, we can recognize that PAD4 inhibitor have important potential therapeutic effects for inflammatory diseases. Third, it has been suggested that there are multiple links in the NETosis that depend on NADPH oxidase (88). Inhibition of NADPH oxidase and blocking NADPH oxidase activation of downstream signaling pathways can reduce the formation of NETs (89). The use of NADPH oxidase inhibitors can block the induction of NETs formation by stimuli and further block the production of profibrotic factors such as TNF-α (90). In addition, plasma membrane rupture is a critical step in the NETosis (91). It has been suggested that PKCα can mediate the phosphorylation of lamin B, which promotes the rupture of the nuclear membrane and thus promotes the formation of NET, and overexpression of nuclear lamin B may reduce the release of NETs (92). Cyclin-dependent kinases 4 and 6 (CDK4/6) regulates the core steps of chromatin decondensation and cleavage in NETosis, and targeted inhibition of CDK4/6 can significantly reduce NETs formation (93).

The core advantages of this NETosis-blocking strategy are as follows. First, aberrant NETs activation in PF acts as a key upstream signal driving fibroblast foci formation and excessive ECM deposition. Blocking NETosis thus directly inhibits the inflammatory-fibrotic vicious circle of PF and delays fibrotic progression. Second, NETs are a pivotal driver of fibroblast activation in low-inflammatory IPF and early-stage PF, and upstream source inhibition of NETosis not only retards fibrotic progression but also avoids the ineffectiveness of intervening in preformed NETs in advanced disease. However, the potential risks of this strategy are non-negligible. PAD4 not only mediates NETosis but also exerts essential roles in neutrophil-mediated antibacterial defense and adaptive immune regulation (94). So its long-term inhibition may increase susceptibility to bacterial and fungal infections, which is particularly pertinent to secondary PF patients with comorbid pulmonary infections (95). As a key antimicrobial enzyme in neutrophils, NE inhibition may impair the airway mucosal defense barrier and elevate the risk of PF acute exacerbations (96). In addition, these inhibitors lack the capacity to clear preformed and deposited NETs, and monotherapy with them yields limited efficacy in patients with advanced PF (97).

#### 5.1.2 Elimination of preformed NETs

DNase can effectively degrade and dissolve the DNA backbone in NETs, and numerous studies have demonstrated that the presence and function of DNase *in vivo* is a key factor in maintaining a low concentration of cell-free DNA (98). Among them, DNase I is the main nuclease present in the inner environment and it cleaves NETs into many small fragments for efficient clearance (98). If NETs are not effectively cleared, multiple components of NETs, such as DNA and histones, will act as immunogens to continuously stimulate the immune response (24). So this process is equally effective and important in mitigating the overactivation of the immune system. In fact, DNase I has been used in the treatment of a variety of inflammatory diseases (99, 100). As PF progresses, a large number of NETs are deposited in the lung tissue, which are cross-linked with the ECM, further exacerbating tissue remodeling. DNases directly degrade DNA components, loosening the ECM network while eliminating the continuous activation signal of NETs to fibroblasts (58). The use of DNase has clear advantages: it targets only extracellular DNA and does not interfere with the normal immune defense functions of neutrophils, thus posing a lower risk of infection compared to PAD4 and NE inhibitors. However, the limitations of DNase in the treatment of pulmonary fibrosis are also evident. First, in the late stages of pulmonary fibrosis, severe lung tissue fibrosis makes it difficult for the DNase administered via nebulization to penetrate the NETs deposits in the pulmonary interstitium, resulting in limited local therapeutic effects (97). Second, while DNase can remove existing NETs, it cannot prevent their formation. But long-term use may carry potential immune risks.

In addition, emerging strategies have emerged in recent years. Recombinant Annexin A1 is a NETs scavenging molecule that has attracted much attention in recent years (101, 102). It induces macrophage phagocytosis of NETs by binding to the phospholipid components of NETs, while inhibiting the release of NETs-related inflammatory factors (103). In PF, the advantages of this strategy are as follows. First, it clears NETs that cross-link with the ECM or bind to cells, especially for intermediate and late PF (103). Second, it has both anti-inflammatory and clearing functions, which can reduce the inflammatory drive for NETs regeneration. At present, this class of drugs is still in the preclinical research stage related to PF, but it has shown a wider clearance profile than DNase, which is the direction for future optimization (103). At the same time, neutralizing antibodies against specific components of NETs (such as Cit-H3) disrupt the structural stability of NETs by specifically binding to the core proteins in NETs, while activating the complement system to mediate NETs clearance (104). The important advantage of this strategy is that it is highly targeted and avoids interference with normal immune function (105). In PF, anti-Cit-H3 antibodies have been shown to reduce NETs deposition in lung tissue while inhibiting fibroblast activation, but currently face clinical translational challenges due to high production costs and intravenous administration (106).

#### 5.1.3 Blockade of NET-associated signaling pathways

Previous studies have shown that the release of NETs is also regulated by a variety of signaling molecules and signaling pathways. Therefore, blocking NETs-related signaling pathways has become a key complementary strategy: directly cutting off the initiated pro-fibrotic signal-driven ECM deposition by targeting the core signaling molecules downstream of NETs that mediate fibrosis.

First, the core components of NETs can directly induce the secretion of TGF-β1 by macrophages and fibroblasts, or release the active TGF-β by degrading TGF-β inhibitors, thereby activating Smad3 phosphorylation and initiating fibroblast activation, myofibroblast differentiation, and ECM synthesis (56, 107). Moreover, TGF-β/Smad3 is the key fibrosis driver pathway of PF, and blocking this pathway can directly inhibit the most critical pathological process of PF without relying on the clearance or production inhibition of NETs (108, 109). Second, NETs activate NF-κB in two ways: on the one hand, they directly bind to the surface TLR4/TLR9 receptor of immune cells, initiate NF-κB nuclear translocation, promote the secretion of pro-inflammatory factors such as IL-6 and IL-1β, and construct a pro-fibrotic inflammatory microenvironment (110). On the other hand, it directly activates the NF-κB pathway in fibroblasts, enhancing their proliferation and ECM synthesis capabilities. This strategy may be adapted to strong inflammation-driven secondary PF (e.g., COPD, asthma-related fibrosis), in which NETs-mediated NF-κB activation is key to the inflammation-fibrotic transition, and blockade can inhibit both inflammatory amplification and fibrotic progression (60, 111). Third, NETs can bind to TLR9 and RAGE receptors on the surface of fibroblasts, which together activate MAPK/ERK signaling in fibroblasts and directly induce fibroblast activation and collagen synthesis (112–114). The TLR9/RAGE pathway is the core pathway for NETs to act directly on fibroblasts, with strong targeting and low off-target effects, making it suitable for IPF patients with significant NETs deposition and low levels of inflammatory factors (115). TLR9 inhibitors directly block NETs-fibroblast binding and avoid sustained activation due to incomplete NETs clearance (60, 114). Finally, NETs can induce Th17 cell differentiation and secretion of IL-17, while histones in NETs can directly activate mast cells to release IL-17 (65, 116). IL-17, in turn, promotes neutrophil activation and NETosis, creating a vicious cycle that continuously drives fibroblast activation and ECM deposition (77, 117, 118).

However, long-term blockade of these signaling pathways may affect normal cell signaling, inhibit the body’s normal anti-inflammatory defense function, and increase the risk of infection in PF patients.

### 5.2 Clinical transformation challenges and optimization directions

Although NET targeting strategies have shown potential in basic research and early clinical practice of PF, there are still multiple common challenges from the laboratory to clinical application, which are not only due to the characteristics of the drug itself, but also closely related to the disease complexity and patient heterogeneity of PF. Based on the existing research evidence, this part will systematically sort out the core conversion obstacles and propose feasible optimization directions.

First, there is currently no uniform NETs-related biomarker for stratification and efficacy assessment in PF patients. Commonly used markers such as cit-H3 and MPO in clinical practice are elevated in PF and other inflammatory lung diseases, lacking disease specificity (14). And these markers cannot judge the patient’s disease stage, resulting in strong blindness in the choice of treatment strategy. In addition, the expression profiles of NETs-related markers for different PF subtypes have not been well determined, further hindering the clinical translation of subtype-specific therapies.

Second, NETs-related signaling pathways (such as TGF-β, NF-κB) and key molecules (such as PAD4 and NE) are involved in normal physiological functions of the body, and most of the existing inhibitors are pan-targets, and long-term use may lead to off-target effects (97). However, PF patients themselves have impaired lung function, fragile immunity, and easy co-infection, and have a lower tolerance to off-target effects.

Third, the fibrosis process of PF is driven by multipathway synergy, and a single NET targeting strategy can easily trigger pathway compensation, resulting in limited clinical benefits of existing strategies in advanced PF and difficult to meet clinical needs (119).

Finally, there are obvious limitations to traditional drug administration. Nebulized inhaled DNase has difficulty penetrating the dense ECM to reach the lung interstitium deposited by NETs, while PF patients are mostly elderly and have poor adherence to long-term intravenous administration (99, 120). These administration-level issues further hinder clinical translation.

Future research should therefore be prioritized in the following aspects. Research should focus on leveraging multi-omics technologies to decode subtype-specific NETosis regulatory circuits, validating multi-parametric NETs-fibrotic biomarker panels through large-scale multi-center cohorts, optimizing and validating subtype-adapted combination therapies in preclinical models and biomarker-stratified clinical trials, and conducting large-scale observational studies to clarify long-term safety risks while establishing personalized monitoring systems, thereby advancing the clinical translation of NET-targeted strategies and promoting precision medicine for PF.

## 6 Conclusion

This review systematically deciphers the subtype-specific regulatory mechanisms of NETosis in IPF and non-IPF-PF, a core novelty that distinguishes it from previous studies which only focused on the general role of NETs in pulmonary fibrosis without addressing pathological heterogeneity across PF subtypes. We further construct a comprehensive regulatory network of NETs in mediating inflammation-fibrosis transition, fibroblast activation and innate-adaptive immune crosstalk, and for the first time conduct a critical comparative analysis of three major NET-targeted therapeutic strategies with a focus on subtype adaptability, filling the gap in existing reviews that lack integrated evaluation of NET-targeted therapies for pulmonary fibrosis. This work not only provides a novel and precise theoretical framework for understanding the immunopathogenic basis of pulmonary fibrosis, but also offers actionable optimization directions for the clinical translation of NET-targeted strategies, laying a solid foundation for the development of subtype-specific precision medicine for pulmonary fibrosis.

### 6.1 Literature search strategy

A systematic literature search was conducted in PubMed and Web of Science from database inception to January 2026, with no initial restrictions to ensure comprehensive coverage. The search combined Medical Subject Headings (MeSH) and free-text terms using Boolean operators (AND/OR), with the core strategy formulated as (neutrophil extracellular traps OR NETs OR NETosis) AND (pulmonary fibrosis OR idiopathic pulmonary fibrosis OR IPF OR silicosis OR interstitial lung disease OR ILD) to align with the review’s focus on NETs in pulmonary fibrosis (PF) and its key subtypes. Studies were included if they were English-language peer-reviewed publications (original basic/clinical research, reviews, meta-analyses, clinical trial data) focusing on NETs-related mechanisms, biomarkers or therapeutic strategies in PF with subtype-specific analysis (IPF/non-IPF-PF). Excluded studies comprised non-English articles, conference abstracts, unpublished data, case reports without mechanistic/therapeutic insights, research unrelated to NETs, *in vitro*/*in vivo* studies without clear PF-related outcomes or subtype stratification, and duplicate publications. Two independent investigators performed literature screening via title/abstract and subsequent full-text assessment, extracted key study data (design, core findings, quantitative results) and conducted cross-validation, with discrepancies resolved by a third senior investigator to minimize selection bias.
